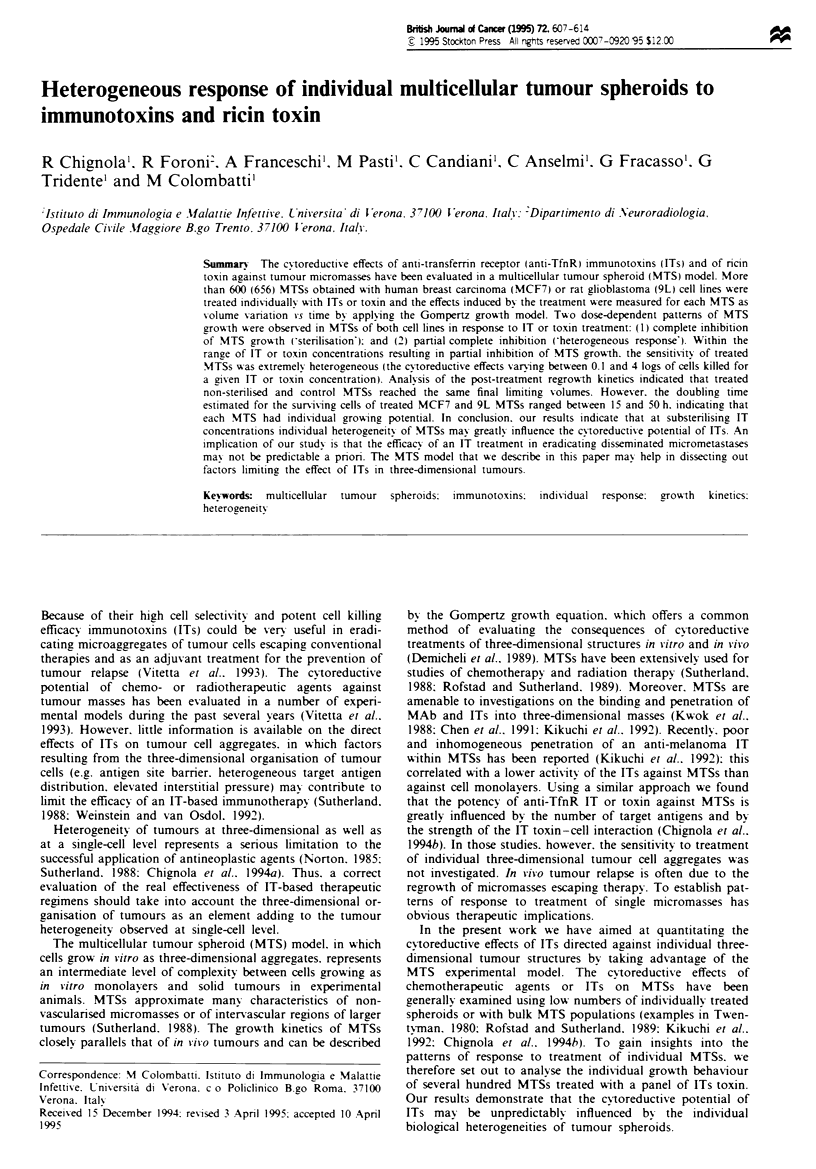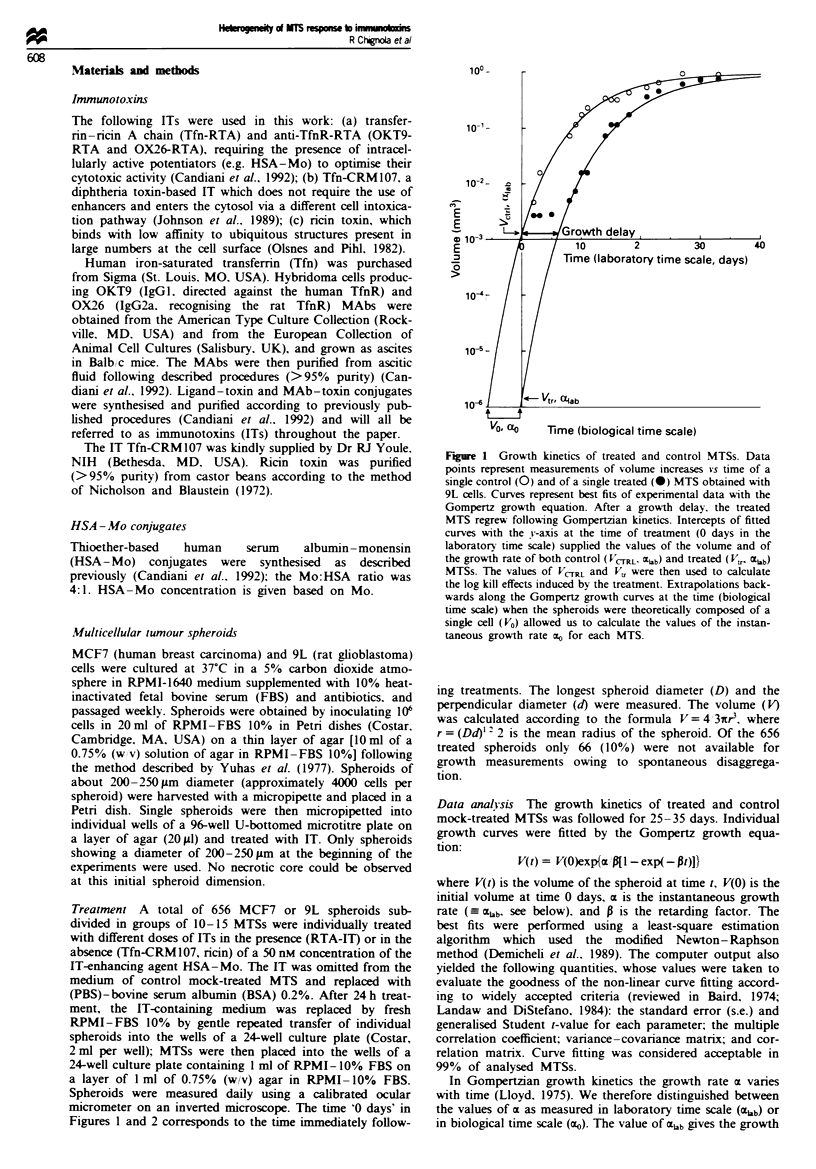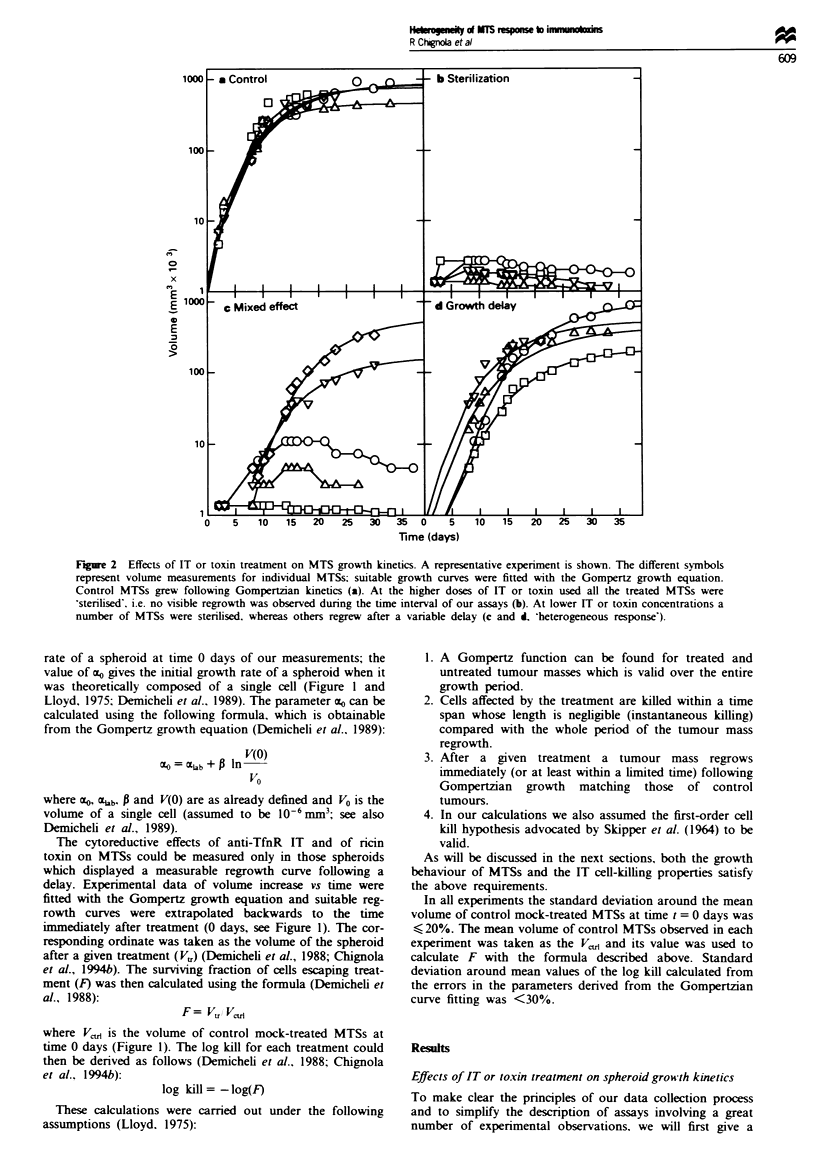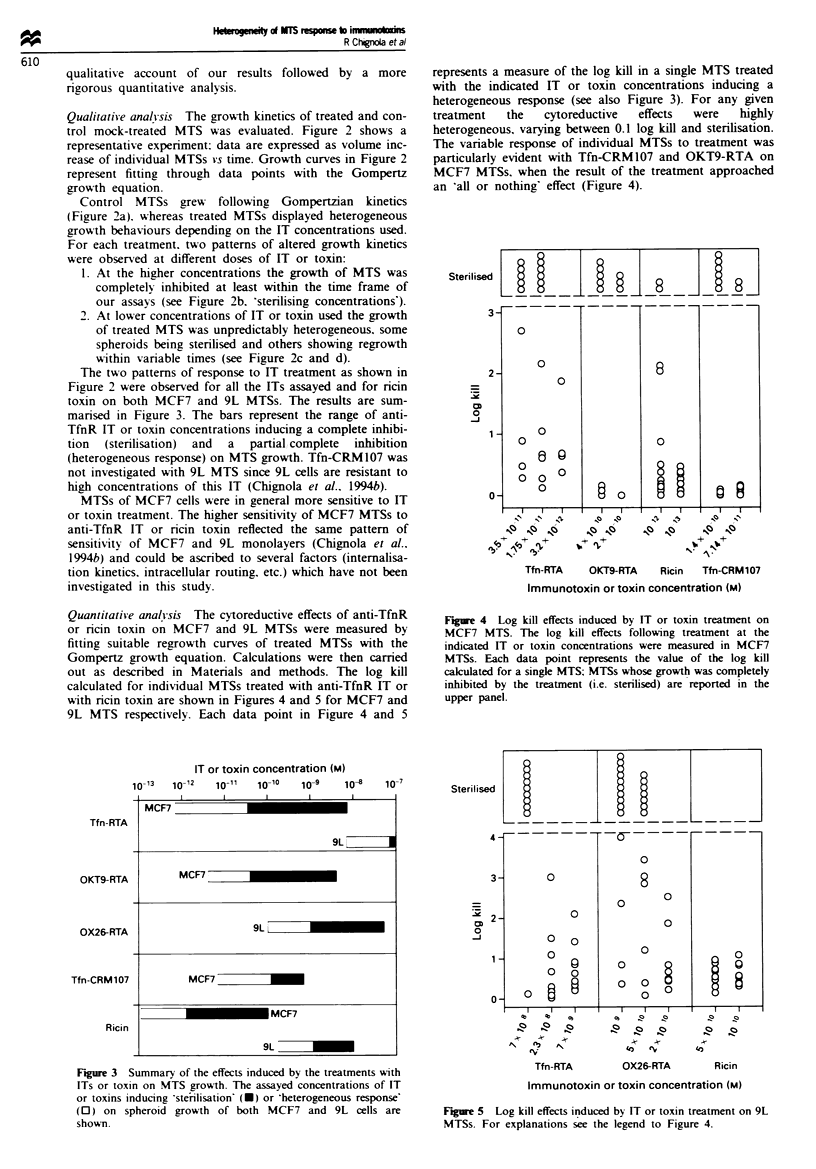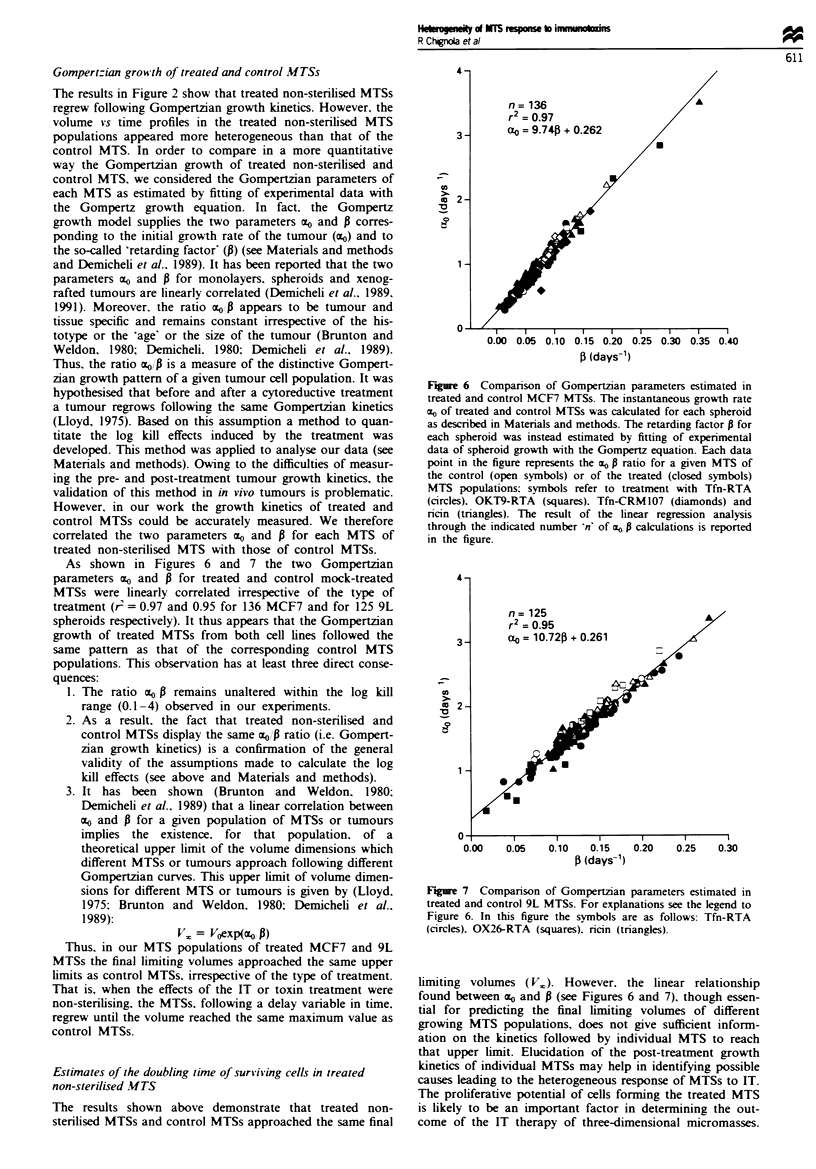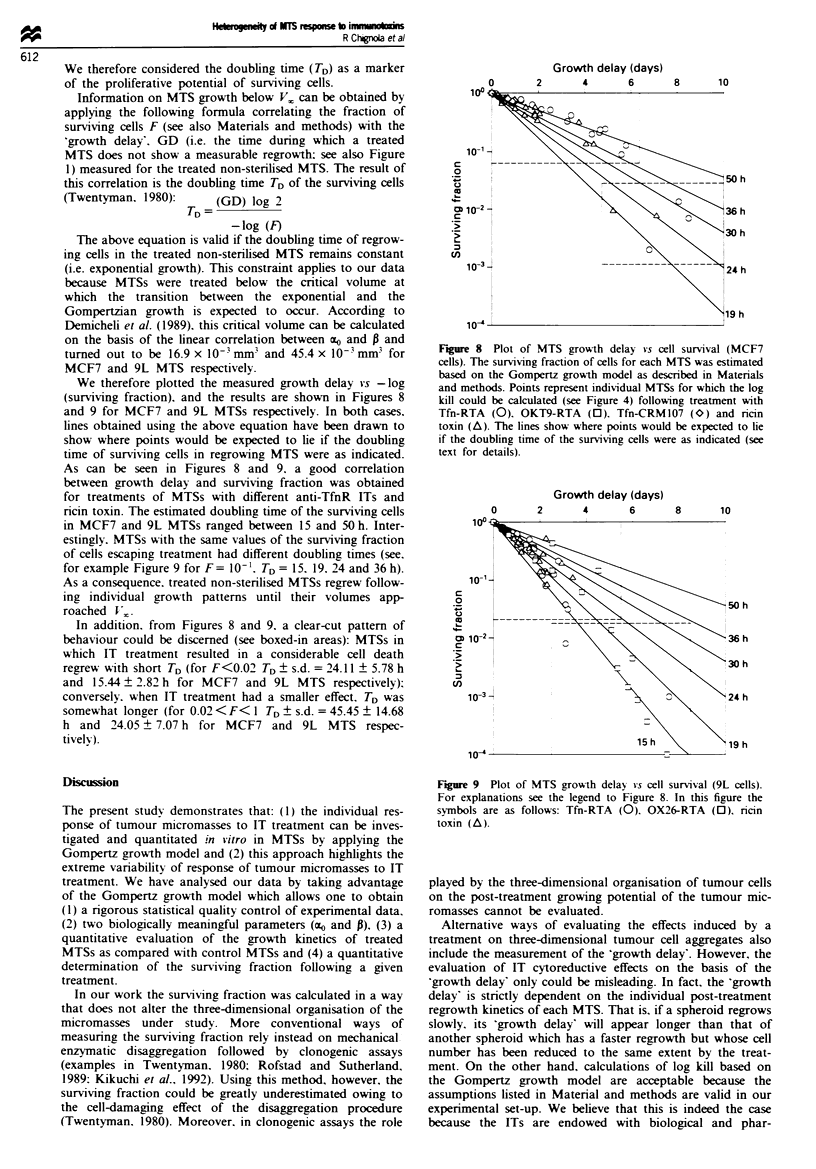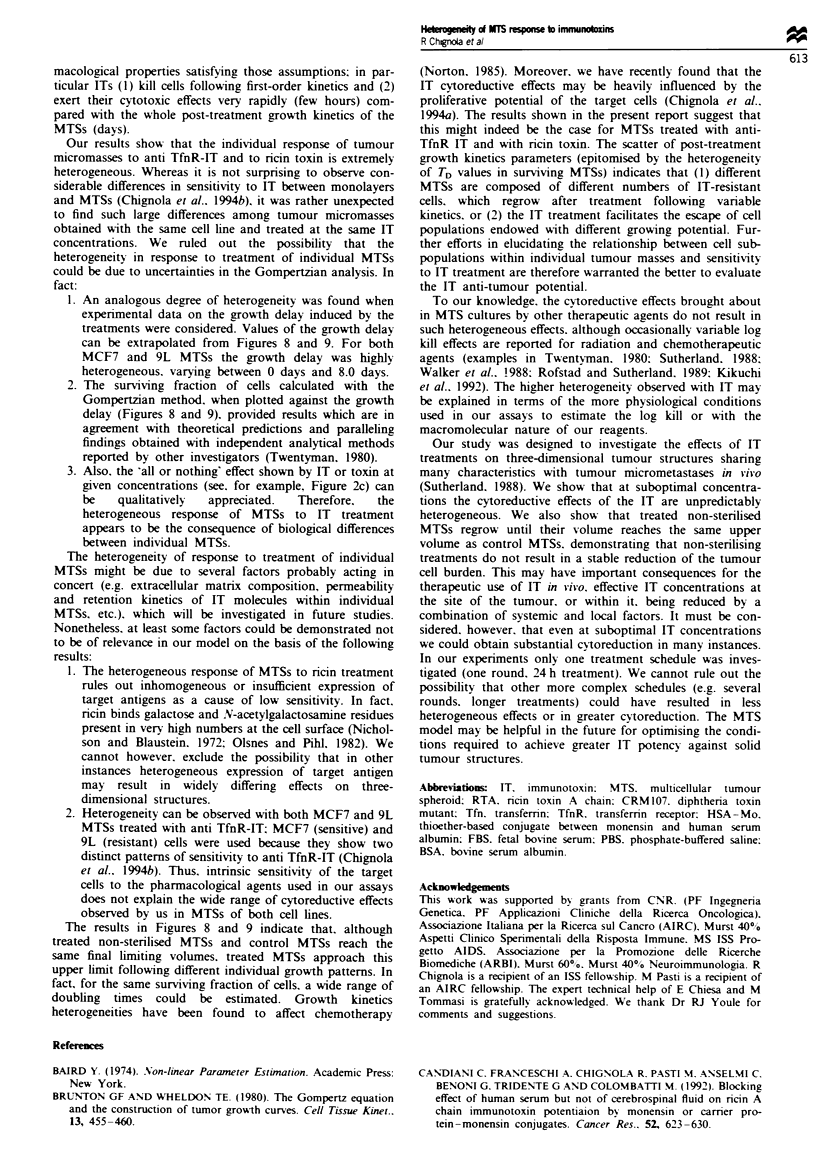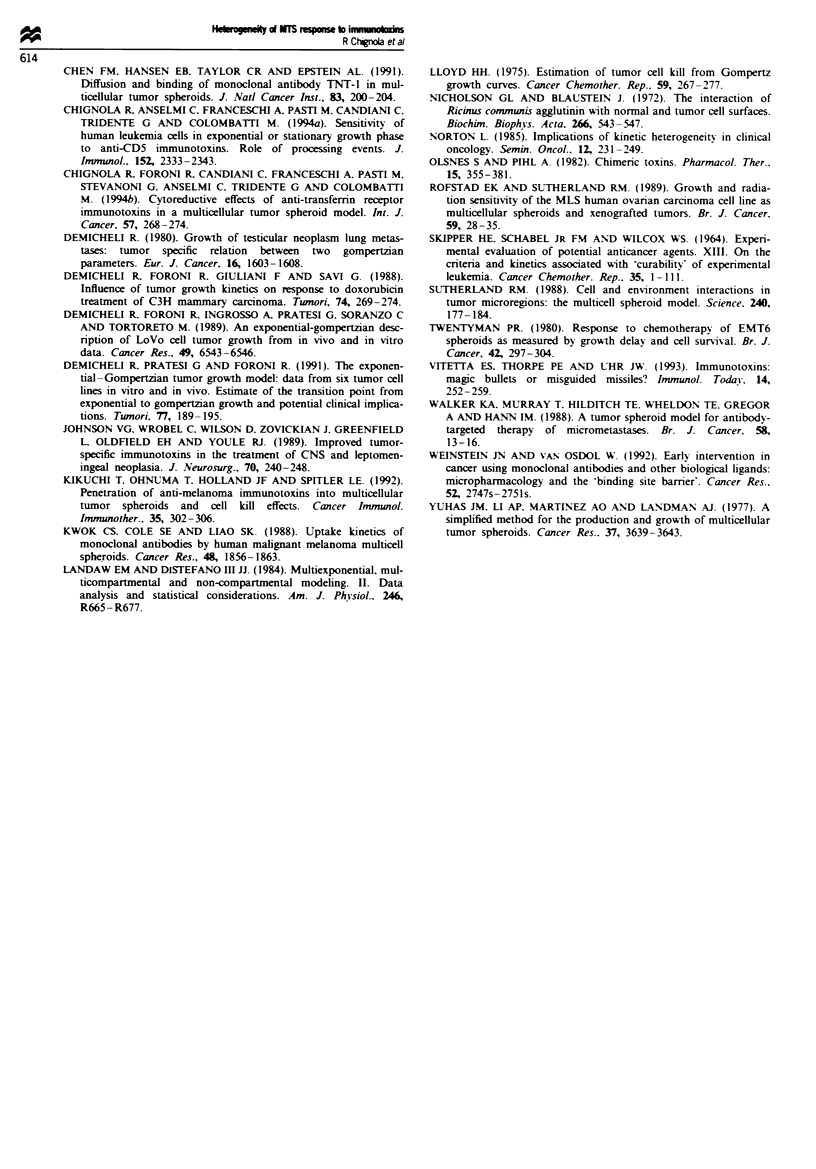# Heterogeneous response of individual multicellular tumour spheroids to immunotoxins and ricin toxin.

**DOI:** 10.1038/bjc.1995.381

**Published:** 1995-09

**Authors:** R. Chignola, R. Foroni, A. Franceschi, M. Pasti, C. Candiani, C. Anselmi, G. Fracasso, G. Tridente, M. Colombatti

**Affiliations:** Istituto di Immunologia e Malattie Infettive, Universita' di Verona, Italy.

## Abstract

The cytoreductive effects of anti-transferrin receptor (anti-TfnR) immunotoxins (ITs) and of ricin toxin against tumour micromasses have been evaluated in a multicellular tumour spheroid (MTS) model. More than 600 (656) MTSs obtained with human breast carcinoma (MCF7) or rat glioblastoma (9L) cell lines were treated individually with ITs or toxin and the effects induced by the treatment were measured for each MTS as volume variation vs time by applying the Gompertz growth model. Two dose-dependent patterns of MTS growth were observed in MTSs of both cell lines in response to IT or toxin treatment: (1) complete inhibition of MTS growth ('sterilisation'); and (2) partial/complete inhibition ('heterogeneous response'). Within the range of IT or toxin concentrations resulting in partial inhibition of MTS growth, the sensitivity of treated MTSs was extremely heterogeneous (the cytoreductive effects varying between 0.1 and 4 logs of cells killed for a given IT or toxin concentration). Analysis of the post-treatment regrowth kinetics indicated that treated non-sterilised and control MTSs reached the same final limiting volumes. However, the doubling time estimated for the surviving cells of treated MCF7 and 9L MTSs ranged between 15 and 50 h, indicating that each MTS had individual growing potential. In conclusion, our results indicate that at substerilising IT concentrations individual heterogenicity of MTSs may greatly influence the cytoreductive potential of ITs. An implication of our study is that the efficacy of an IT treatment in eradicating disseminated micrometastases may not be predictable a priori. The MTS model that we describe in this paper may help in dissecting out factors limiting the effect of ITs in three-dimensional tumours.


British Journal of Cancer (1995) 72, 607-614

© 1995 Stockton Press All rights reserved 0007-0920/95 $12.00

### Heterogeneous response of individual multicellular tumour spheroids to immunotoxins and ricin toxin

R Chignola[1], R Foroni[2], A Franceschi[1], M Pasti[1], C Candiani[1], C Anselmi[1], G Fracasso[1], G Tridente[1] and M Colombatti[1]

[1]*Istituto di Immunologia e Malattie Infettive, Universita' di Verona, 37100 Verona, Italy;* [2]*Dipartimento di Neuroradiologia, Ospedale Civile Maggiore B.go Trento, 37100 Verona, Italy.*

**Summary** The cytoreductive effects of anti-transferrin receptor (anti-TfnR) immunotoxins (ITs) and of ricin toxin against tumour micromasses have been evaluated in a multicellular tumour spheroid (MTS) model. More than 600 (656) MTSs obtained with human breast carcinoma (MCF7) or rat glioblastoma (9L) cell lines were treated individually with ITs or toxin and the effects induced by the treatment were measured for each MTS as volume variation *vs* time by applying the Gompertz growth model. Two dose-dependent patterns of MTS growth were observed in MTSs of both cell lines in response to IT or toxin treatment: (1) complete inhibition of MTS growth ('sterilisation'); and (2) partial complete inhibition ('heterogeneous response'). Within the range of IT or toxin concentrations resulting in partial inhibition of MTS growth, the sensitivity of treated MTSs was extremely heterogeneous (the cytoreductive effects varying between 0.1 and 4 logs of cells killed for a given IT or toxin concentration). Analysis of the post-treatment regrowth kinetics indicated that treated non-sterilised and control MTSs reached the same final limiting volumes. However, the doubling time estimated for the surviving cells of treated MCF7 and 9L MTSs ranged between 15 and 50 h, indicating that each MTS had individual growing potential. In conclusion, our results indicate that at substerilising IT concentrations individual heterogeneity of MTSs may greatly influence the cytoreductive potential of ITs. An implication of our study is that the efficacy of an IT treatment in eradicating disseminated micrometastases may not be predictable a priori. The MTS model that we describe in this paper may help in dissecting out factors limiting the effect of ITs in three-dimensional tumours.

**Keywords:** multicellular tumour spheroids; immunotoxins; individual response; growth kinetics; heterogeneity

Because of their high cell selectivity and potent cell killing efficacy immunotoxins (ITs) could be very useful in eradicating microaggregates of tumour cells escaping conventional therapies and as an adjuvant treatment for the prevention of tumour relapse (Vitetta *et al.*, 1993). The cytoreductive potential of chemo- or radiotherapeutic agents against tumour masses has been evaluated in a number of experimental models during the past several years (Vitetta *et al.*, 1993). However, little information is available on the direct effects of ITs on tumour cell aggregates, in which factors resulting from the three-dimensional organisation of tumour cells (e.g. antigen site barrier, heterogeneous target antigen distribution, elevated interstitial pressure) may contribute to limit the efficacy of an IT-based immunotherapy (Sutherland, 1988; Weinstein and van Osdol, 1992).

Heterogeneity of tumours at three-dimensional as well as at a single-cell level represents a serious limitation to the successful application of antineoplastic agents (Norton, 1985; Sutherland, 1988; Chignola *et al.*, 1994*a*). Thus, a correct evaluation of the real effectiveness of IT-based therapeutic regimens should take into account the three-dimensional organisation of tumours as an element adding to the tumour heterogeneity observed at single-cell level.

The multicellular tumour spheroid (MTS) model, in which cells grow *in vitro* as three-dimensional aggregates, represents an intermediate level of complexity between cells growing as *in vitro* monolayers and solid tumours in experimental animals. MTSs approximate many characteristics of non-vascularised micromasses or of intervascular regions of larger tumours (Sutherland, 1988). The growth kinetics of MTSs closely parallels that of *in vivo* tumours and can be described by the Gompertz growth equation, which offers a common method of evaluating the consequences of cytoreductive treatments of three-dimensional structures *in vitro* and *in vivo* (Demicheli *et al.*, 1989). MTSs have been extensively used for studies of chemotherapy and radiation therapy (Sutherland, 1988; Rofstad and Sutherland, 1989). Moreover, MTSs are amenable to investigations on the binding and penetration of MAb and ITs into three-dimensional masses (Kwok *et al.*, 1988; Chen *et al.*, 1991; Kikuchi *et al.*, 1992). Recently, poor and inhomogeneous penetration of an anti-melanoma IT within MTSs has been reported (Kikuchi *et al.*, 1992); this correlated with a lower activity of the ITs against MTSs than against cell monolayers. Using a similar approach we found that the potency of anti-TfnR IT or toxin against MTSs is greatly influenced by the number of target antigens and by the strength of the IT toxin-cell interaction (Chignola *et al.*, 1994*b*). In those studies, however, the sensitivity to treatment of individual three-dimensional tumour cell aggregates was not investigated. *In vivo* tumour relapse is often due to the regrowth of micromasses escaping therapy. To establish patterns of response to treatment of single micromasses has obvious therapeutic implications.

In the present work we have aimed at quantitating the cytoreductive effects of ITs directed against individual three-dimensional tumour structures by taking advantage of the MTS experimental model. The cytoreductive effects of chemotherapeutic agents or ITs on MTSs have been generally examined using low numbers of individually treated spheroids or with bulk MTS populations (examples in Twentyman, 1980; Rofstad and Sutherland, 1989; Kikuchi *et al.*, 1992; Chignola *et al.*, 1994*b*). To gain insights into the patterns of response to treatment of individual MTSs, we therefore set out to analyse the individual growth behaviour of several hundred MTSs treated with a panel of ITs toxin. Our results demonstrate that the cytoreductive potential of ITs may be unpredictably influenced by the individual biological heterogeneities of tumour spheroids.

Correspondence: M Colombatti, Istituto di Immunologia e Malattie Infettive, Università di Verona, c/o Policlinico B.go Roma, 37100 Verona, Italy

Received 15 December 1994; revised 3 April 1995; accepted 10 April 1995

### Materials and methods

### *Immunotoxins*

The following ITs were used in this work: (a) transferrin–ricin A chain (Tfn-RTA) and anti-TfnR-RTA (OKT9-RTA and OX26-RTA), requiring the presence of intracellularly active potentiators (e.g. HSA–Mo) to optimise their cytotoxic activity (Candiani *et al.*, 1992); (b) Tfn-CRM107, a diphtheria toxin-based IT which does not require the use of enhancers and enters the cytosol via a different cell intoxication pathway (Johnson *et al.*, 1989); (c) ricin toxin, which binds with low affinity to ubiquitous structures present in large numbers at the cell surface (Olsnes and Pihl, 1982).

Human iron-saturated transferrin (Tfn) was purchased from Sigma (St. Louis, MO, USA). Hybridoma cells producing OKT9 (IgG1, directed against the human TfnR) and OX26 (IgG2a, recognising the rat TfnR) MAbs were obtained from the American Type Culture Collection (Rockville, MD, USA) and from the European Collection of Animal Cell Cultures (Salisbury, UK), and grown as ascites in Balb/c mice. The MAbs were then purified from ascitic fluid following described procedures (>95% purity) (Candiani *et al.*, 1992). Ligand–toxin and MAb–toxin conjugates were synthesised and purified according to previously published procedures (Candiani *et al.*, 1992) and will all be referred to as immunotoxins (ITs) throughout the paper.

The IT Tfn-CRM107 was kindly supplied by Dr RJ Youle, NIH (Bethesda, MD, USA). Ricin toxin was purified (>95% purity) from castor beans according to the method of Nicholson and Blaustein (1972).

### *HSA–Mo conjugates*

Thioether-based human serum albumin–monensin (HSA–Mo) conjugates were synthesised as described previously (Candiani *et al.*, 1992); the Mo:HSA ratio was 4:1. HSA–Mo concentration is given based on Mo.

### *Multicellular tumour spheroids*

MCF7 (human breast carcinoma) and 9L (rat glioblastoma) cells were cultured at 37°C in a 5% carbon dioxide atmosphere in RPMI-1640 medium supplemented with 10% heat-inactivated fetal bovine serum (FBS) and antibiotics, and passaged weekly. Spheroids were obtained by inoculating $10^6$ cells in 20 ml of RPMI–FBS 10% in Petri dishes (Costar, Cambridge, MA, USA) on a thin layer of agar [10 ml of a 0.75% (w/v) solution of agar in RPMI–FBS 10%] following the method described by Yuhas *et al.* (1977). Spheroids of about 200–250 μm diameter (approximately 4000 cells per spheroid) were harvested with a micropipette and placed in a Petri dish. Single spheroids were then micropipetted into individual wells of a 96-well U-bottomed microtitre plate on a layer of agar (20 μl) and treated with IT. Only spheroids showing a diameter of 200–250 μm at the beginning of the experiments were used. No necrotic core could be observed at this initial spheroid dimension.

*Treatment* A total of 656 MCF7 or 9L spheroids subdivided in groups of 10–15 MTSs were individually treated with different doses of ITs in the presence (RTA-IT) or in the absence (Tfn-CRM107, ricin) of a 50 nM concentration of the IT-enhancing agent HSA–Mo. The IT was omitted from the medium of control mock-treated MTS and replaced with (PBS)–bovine serum albumin (BSA) 0.2%. After 24 h treatment, the IT-containing medium was replaced by fresh RPMI–FBS 10% by gentle repeated transfer of individual spheroids into the wells of a 24-well culture plate (Costar, 2 ml per well); MTSs were then placed into the wells of a 24-well culture plate containing 1 ml of RPMI–10% FBS on a layer of 1 ml of 0.75% (w/v) agar in RPMI–10% FBS. Spheroids were measured daily using a calibrated ocular micrometer on an inverted microscope. The time '0 days' in Figures 1 and 2 corresponds to the time immediately following treatments. The longest spheroid diameter ($D$) and the perpendicular diameter ($d$) were measured. The volume ($V$) was calculated according to the formula $V = 4/3\pi r^3$, where $r = (Dd)^{1/2}/2$ is the mean radius of the spheroid. Of the 656 treated spheroids only 66 (10%) were not available for growth measurements owing to spontaneous disaggregation.

**Figure 1** Growth kinetics of treated and control MTSs. Data points represent measurements of volume increases *vs* time of a single control (○) and of a single treated (●) MTS obtained with 9L cells. Curves represent best fits of experimental data with the Gompertz growth equation. After a growth delay, the treated MTS regrew following Gompertzian kinetics. Intercepts of fitted curves with the *y*-axis at the time of treatment (0 days in the laboratory time scale) supplied the values of the volume and of the growth rate of both control ($V_{CTRL}$, $\alpha_{lab}$) and treated ($V_{tr}$, $\alpha_{lab}$) MTSs. The values of $V_{CTRL}$ and $V_{tr}$ were then used to calculate the log kill effects induced by the treatment. Extrapolations backwards along the Gompertz growth curves at the time (biological time scale) when the spheroids were theoretically composed of a single cell ($V_0$) allowed us to calculate the values of the instantaneous growth rate $\alpha_0$ for each MTS.

*Data analysis* The growth kinetics of treated and control mock-treated MTSs was followed for 25–35 days. Individual growth curves were fitted by the Gompertz growth equation:

$$V(t) = V(0)\exp\{\alpha/\beta[1 - \exp(-\beta t)]\}$$

where $V(t)$ is the volume of the spheroid at time $t$, $V(0)$ is the initial volume at time 0 days, $\alpha$ is the instantaneous growth rate ($\equiv \alpha_{lab}$, see below), and $\beta$ is the retarding factor. The best fits were performed using a least-square estimation algorithm which used the modified Newton–Raphson method (Demicheli *et al.*, 1989). The computer output also yielded the following quantities, whose values were taken to evaluate the goodness of the non-linear curve fitting according to widely accepted criteria (reviewed in Baird, 1974; Landaw and DiStefano, 1984): the standard error (s.e.) and generalised Student $t$-value for each parameter; the multiple correlation coefficient; variance–covariance matrix; and correlation matrix. Curve fitting was considered acceptable in 99% of analysed MTSs.

In Gompertzian growth kinetics the growth rate $\alpha$ varies with time (Lloyd, 1975). We therefore distinguished between the values of $\alpha$ as measured in laboratory time scale ($\alpha_{lab}$) or in biological time scale ($\alpha_0$). The value of $\alpha_{lab}$ gives the growth

**Figure 2** Effects of IT or toxin treatment on MTS growth kinetics. A representative experiment is shown. The different symbols represent volume measurements for individual MTSs; suitable growth curves were fitted with the Gompertz growth equation. Control MTSs grew following Gompertzian kinetics (**a**). At the higher doses of IT or toxin used all the treated MTSs were 'sterilised', i.e. no visible regrowth was observed during the time interval of our assays (**b**). At lower IT or toxin concentrations a number of MTSs were sterilised, whereas others regrew after a variable delay (**c** and **d**, 'heterogeneous response').

rate of a spheroid at time 0 days of our measurements; the value of $\alpha_0$ gives the initial growth rate of a spheroid when it was theoretically composed of a single cell (Figure 1 and Lloyd, 1975; Demicheli *et al.*, 1989). The parameter $\alpha_0$ can be calculated using the following formula, which is obtainable from the Gompertz growth equation (Demicheli *et al.*, 1989):

$$\alpha_0 = \alpha_{lab} + \beta \ \ln\frac{V(0)}{V_0}$$

where $\alpha_0$, $\alpha_{lab}$, $\beta$ and $V(0)$ are as already defined and $V_0$ is the volume of a single cell (assumed to be $10^{-6}$ mm$^3$; see also Demicheli *et al.*, 1989).

The cytoreductive effects of anti-TfnR IT and of ricin toxin on MTSs could be measured only in those spheroids which displayed a measurable regrowth curve following a delay. Experimental data of volume increase *vs* time were fitted with the Gompertz growth equation and suitable regrowth curves were extrapolated backwards to the time immediately after treatment (0 days, see Figure 1). The corresponding ordinate was taken as the volume of the spheroid after a given treatment ($V_{tr}$) (Demicheli *et al.*, 1988; Chignola *et al.*, 1994*b*). The surviving fraction of cells escaping treatment ($F$) was then calculated using the formula (Demicheli *et al.*, 1988):

$$F = V_{tr}/V_{ctrl}$$

where $V_{ctrl}$ is the volume of control mock-treated MTSs at time 0 days (Figure 1). The log kill for each treatment could then be derived as follows (Demicheli *et al.*, 1988; Chignola *et al.*, 1994*b*):

$$\log \text{kill} = -\log(F)$$

These calculations were carried out under the following assumptions (Lloyd, 1975):

1. A Gompertz function can be found for treated and untreated tumour masses which is valid over the entire growth period.
2. Cells affected by the treatment are killed within a time span whose length is negligible (instantaneous killing) compared with the whole period of the tumour mass regrowth.
3. After a given treatment a tumour mass regrows immediately (or at least within a limited time) following Gompertzian growth matching those of control tumours.
4. In our calculations we also assumed the first-order cell kill hypothesis advocated by Skipper *et al.* (1964) to be valid.

As will be discussed in the next sections, both the growth behaviour of MTSs and the IT cell-killing properties satisfy the above requirements.

In all experiments the standard deviation around the mean volume of control mock-treated MTSs at time $t = 0$ days was $\leqslant 20\%$. The mean volume of control MTSs observed in each experiment was taken as the $V_{ctrl}$ and its value was used to calculate $F$ with the formula described above. Standard deviation around mean values of the log kill calculated from the errors in the parameters derived from the Gompertzian curve fitting was $<30\%$.

### Results

### *Effects of IT or toxin treatment on spheroid growth kinetics*

To make clear the principles of our data collection process and to simplify the description of assays involving a great number of experimental observations, we will first give a qualitative account of our results followed by a more rigorous quantitative analysis.

*Qualitative analysis* The growth kinetics of treated and control mock-treated MTS was evaluated. Figure 2 shows a representative experiment: data are expressed as volume increase of individual MTSs *vs* time. Growth curves in Figure 2 represent fitting through data points with the Gompertz growth equation.

Control MTSs grew following Gompertzian kinetics (Figure 2a), whereas treated MTSs displayed heterogeneous growth behaviours depending on the IT concentrations used. For each treatment, two patterns of altered growth kinetics were observed at different doses of IT or toxin:

1. At the higher concentrations the growth of MTS was completely inhibited at least within the time frame of our assays (see Figure 2b, 'sterilising concentrations').
2. At lower concentrations of IT or toxin used the growth of treated MTS was unpredictably heterogeneous, some spheroids being sterilised and others showing regrowth within variable times (see Figure 2c and d).

The two patterns of response to IT treatment as shown in Figure 2 were observed for all the ITs assayed and for ricin toxin on both MCF7 and 9L MTSs. The results are summarised in Figure 3. The bars represent the range of anti-TfnR IT or toxin concentrations inducing a complete inhibition (sterilisation) and a partial/complete inhibition (heterogeneous response) on MTS growth. Tfn-CRM107 was not investigated with 9L MTS since 9L cells are resistant to high concentrations of this IT (Chignola *et al*., 1994*b*).

MTSs of MCF7 cells were in general more sensitive to IT or toxin treatment. The higher sensitivity of MCF7 MTSs to anti-TfnR IT or ricin toxin reflected the same pattern of sensitivity of MCF7 and 9L monolayers (Chignola *et al*., 1994*b*) and could be ascribed to several factors (internalisation kinetics, intracellular routing, etc.) which have not been investigated in this study.

*Quantitative analysis* The cytoreductive effects of anti-TfnR or ricin toxin on MCF7 and 9L MTSs were measured by fitting suitable regrowth curves of treated MTSs with the Gompertz growth equation. Calculations were then carried out as described in Materials and methods. The log kill calculated for individual MTSs treated with anti-TfnR IT or with ricin toxin are shown in Figures 4 and 5 for MCF7 and 9L MTS respectively. Each data point in Figure 4 and 5 represents a measure of the log kill in a single MTS treated with the indicated IT or toxin concentrations inducing a heterogeneous response (see also Figure 3). For any given treatment the cytoreductive effects were highly heterogeneous, varying between 0.1 log kill and sterilisation. The variable response of individual MTSs to treatment was particularly evident with Tfn-CRM107 and OKT9-RTA on MCF7 MTSs, when the result of the treatment approached an 'all or nothing' effect (Figure 4).

**Figure 4** Log kill effects induced by IT or toxin treatment on MCF7 MTS. The log kill effects following treatment at the indicated IT or toxin concentrations were measured in MCF7 MTSs. Each data point represents the value of the log kill calculated for a single MTS; MTSs whose growth was completely inhibited by the treatment (i.e. sterilised) are reported in the upper panel.

**Figure 3** Summary of the effects induced by the treatments with ITs or toxin on MTS growth. The assayed concentrations of IT or toxins inducing 'sterilisation' (■) or 'heterogeneous response' (□) on spheroid growth of both MCF7 and 9L cells are shown.

**Figure 5** Log kill effects induced by IT or toxin treatment on 9L MTSs. For explanations see the legend to Figure 4.

*Gompertzian growth of treated and control MTSs*

The results in Figure 2 show that treated non-sterilised MTSs regrew following Gompertzian growth kinetics. However, the volume *vs* time profiles in the treated non-sterilised MTS populations appeared more heterogeneous than that of the control MTS. In order to compare in a more quantitative way the Gompertzian growth of treated non-sterilised and control MTS, we considered the Gompertzian parameters of each MTS as estimated by fitting of experimental data with the Gompertz growth equation. In fact, the Gompertz growth model supplies the two parameters $\alpha_0$ and $\beta$ corresponding to the initial growth rate of the tumour ($\alpha_0$) and to the so-called 'retarding factor' ($\beta$) (see Materials and methods and Demicheli *et al.*, 1989). It has been reported that the two parameters $\alpha_0$ and $\beta$ for monolayers, spheroids and xenografted tumours are linearly correlated (Demicheli *et al.*, 1989, 1991). Moreover, the ratio $\alpha_0/\beta$ appears to be tumour and tissue specific and remains constant irrespective of the histotype or the 'age' or the size of the tumour (Brunton and Weldon, 1980; Demicheli, 1980; Demicheli *et al.*, 1989). Thus, the ratio $\alpha_0/\beta$ is a measure of the distinctive Gompertzian growth pattern of a given tumour cell population. It was hypothesised that before and after a cytoreductive treatment a tumour regrows following the same Gompertzian kinetics (Lloyd, 1975). Based on this assumption a method to quantitate the log kill effects induced by the treatment was developed. This method was applied to analyse our data (see Materials and methods). Owing to the difficulties of measuring the pre- and post-treatment tumour growth kinetics, the validation of this method in *in vivo* tumours is problematic. However, in our work the growth kinetics of treated and control MTSs could be accurately measured. We therefore correlated the two parameters $\alpha_0$ and $\beta$ for each MTS of treated non-sterilised MTS with those of control MTSs.

As shown in Figures 6 and 7 the two Gompertzian parameters $\alpha_0$ and $\beta$ for treated and control mock-treated MTSs were linearly correlated irrespective of the type of treatment ($r^2 = 0.97$ and 0.95 for 136 MCF7 and for 125 9L spheroids respectively). It thus appears that the Gompertzian growth of treated MTSs from both cell lines followed the same pattern as that of the corresponding control MTS populations. This observation has at least three direct consequences:

1. The ratio $\alpha_0/\beta$ remains unaltered within the log kill range (0.1–4) observed in our experiments.
2. As a result, the fact that treated non-sterilised and control MTSs display the same $\alpha_0/\beta$ ratio (i.e. Gompertzian growth kinetics) is a confirmation of the general validity of the assumptions made to calculate the log kill effects (see above and Materials and methods).
3. It has been shown (Brunton and Weldon, 1980; Demicheli *et al.*, 1989) that a linear correlation between $\alpha_0$ and $\beta$ for a given population of MTSs or tumours implies the existence, for that population, of a theoretical upper limit of the volume dimensions which different MTSs or tumours approach following different Gompertzian curves. This upper limit of volume dimensions for different MTS or tumours is given by (Lloyd, 1975; Brunton and Weldon, 1980; Demicheli *et al.*, 1989):

$$V_\infty = V_0 \exp(\alpha_0/\beta)$$

Thus, in our MTS populations of treated MCF7 and 9L MTSs the final limiting volumes approached the same upper limits as control MTSs, irrespective of the type of treatment. That is, when the effects of the IT or toxin treatment were non-sterilising, the MTSs, following a delay variable in time, regrew until the volume reached the same maximum value as control MTSs.

*Estimates of the doubling time of surviving cells in treated non-sterilised MTS*

The results shown above demonstrate that treated non-sterilised MTSs and control MTSs approached the same final limiting volumes ($V_\infty$). However, the linear relationship found between $\alpha_0$ and $\beta$ (see Figures 6 and 7), though essential for predicting the final limiting volumes of different growing MTS populations, does not give sufficient information on the kinetics followed by individual MTS to reach that upper limit. Elucidation of the post-treatment growth kinetics of individual MTSs may help in identifying possible causes leading to the heterogeneous response of MTSs to IT. The proliferative potential of cells forming the treated MTS is likely to be an important factor in determining the outcome of the IT therapy of three-dimensional micromasses.

**Figure 6** Comparison of Gompertzian parameters estimated in treated and control MCF7 MTSs. The instantaneous growth rate $\alpha_0$ of treated and control MTSs was calculated for each spheroid as described in Materials and methods. The retarding factor $\beta$ for each spheroid was instead estimated by fitting of experimental data of spheroid growth with the Gompertz equation. Each data point in the figure represents the $\alpha_0/\beta$ ratio for a given MTS of the control (open symbols) or of the treated (closed symbols) MTS populations; symbols refer to treatment with Tfn-RTA (circles), OKT9-RTA (squares), Tfn-CRM107 (diamonds) and ricin (triangles). The result of the linear regression analysis through the indicated number '*n*' of $\alpha_0/\beta$ calculations is reported in the figure.

**Figure 7** Comparison of Gompertzian parameters estimated in treated and control 9L MTSs. For explanations see the legend to Figure 6. In this figure the symbols are as follows: Tfn-RTA (circles), OX26-RTA (squares), ricin (triangles).

We therefore considered the doubling time ($T_D$) as a marker of the proliferative potential of surviving cells.

Information on MTS growth below $V_\infty$ can be obtained by applying the following formula correlating the fraction of surviving cells $F$ (see also Materials and methods) with the 'growth delay', GD (i.e. the time during which a treated MTS does not show a measurable regrowth; see also Figure 1) measured for the treated non-sterilised MTS. The result of this correlation is the doubling time $T_D$ of the surviving cells (Twentyman, 1980):

$$T_D = \frac{(GD) \log 2}{-\log (F)}$$

The above equation is valid if the doubling time of regrowing cells in the treated non-sterilised MTS remains constant (i.e. exponential growth). This constraint applies to our data because MTSs were treated below the critical volume at which the transition between the exponential and the Gompertzian growth is expected to occur. According to Demicheli *et al.* (1989), this critical volume can be calculated on the basis of the linear correlation between $\alpha_0$ and $\beta$ and turned out to be $16.9 \times 10^{-3}$ mm$^3$ and $45.4 \times 10^{-3}$ mm$^3$ for MCF7 and 9L MTS respectively.

We therefore plotted the measured growth delay *vs* −log (surviving fraction), and the results are shown in Figures 8 and 9 for MCF7 and 9L MTSs respectively. In both cases, lines obtained using the above equation have been drawn to show where points would be expected to lie if the doubling time of surviving cells in regrowing MTS were as indicated. As can be seen in Figures 8 and 9, a good correlation between growth delay and surviving fraction was obtained for treatments of MTSs with different anti-TfnR ITs and ricin toxin. The estimated doubling time of the surviving cells in MCF7 and 9L MTSs ranged between 15 and 50 h. Interestingly, MTSs with the same values of the surviving fraction of cells escaping treatment had different doubling times (see, for example Figure 9 for $F = 10^{-1}$, $T_D$ = 15, 19, 24 and 36 h). As a consequence, treated non-sterilised MTSs regrew following individual growth patterns until their volumes approached $V_\infty$.

In addition, from Figures 8 and 9, a clear-cut pattern of behaviour could be discerned (see boxed-in areas): MTSs in which IT treatment resulted in a considerable cell death regrew with short $T_D$ (for $F<0.02$ $T_D \pm$ s.d. = 24.11 ± 5.78 h and 15.44 ± 2.82 h for MCF7 and 9L MTS respectively); conversely, when IT treatment had a smaller effect, $T_D$ was somewhat longer (for $0.02<F<1$ $T_D \pm$ s.d. = 45.45 ± 14.68 h and 24.05 ± 7.07 h for MCF7 and 9L MTS respectively).

**Figure 8** Plot of MTS growth delay *vs* cell survival (MCF7 cells). The surviving fraction of cells for each MTS was estimated based on the Gompertz growth model as described in Materials and methods. Points represent individual MTSs for which the log kill could be calculated (see Figure 4) following treatment with Tfn-RTA (○), OKT9-RTA (□), Tfn-CRM107 (◇) and ricin toxin (△). The lines show where points would be expected to lie if the doubling time of the surviving cells were as indicated (see text for details).

**Figure 9** Plot of MTS growth delay *vs* cell survival (9L cells). For explanations see the legend to Figure 8. In this figure the symbols are as follows: Tfn-RTA (○), OX26-RTA (□), ricin toxin (△).

### Discussion

The present study demonstrates that: (1) the individual response of tumour micromasses to IT treatment can be investigated and quantitated *in vitro* in MTSs by applying the Gompertz growth model and (2) this approach highlights the extreme variability of response of tumour micromasses to IT treatment. We have analysed our data by taking advantage of the Gompertz growth model which allows one to obtain (1) a rigorous statistical quality control of experimental data, (2) two biologically meaningful parameters ($\alpha_0$ and $\beta$), (3) a quantitative evaluation of the growth kinetics of treated MTSs as compared with control MTSs and (4) a quantitative determination of the surviving fraction following a given treatment.

In our work the surviving fraction was calculated in a way that does not alter the three-dimensional organisation of the micromasses under study. More conventional ways of measuring the surviving fraction rely instead on mechanical enzymatic disaggregation followed by clonogenic assays (examples in Twentyman, 1980; Rofstad and Sutherland, 1989; Kikuchi *et al.*, 1992). Using this method, however, the surviving fraction could be greatly underestimated owing to the cell-damaging effect of the disaggregation procedure (Twentyman, 1980). Moreover, in clonogenic assays the role played by the three-dimensional organisation of tumour cells on the post-treatment growing potential of the tumour micromasses cannot be evaluated.

Alternative ways of evaluating the effects induced by a treatment on three-dimensional tumour cell aggregates also include the measurement of the 'growth delay'. However, the evaluation of IT cytoreductive effects on the basis of the 'growth delay' only could be misleading. In fact, the 'growth delay' is strictly dependent on the individual post-treatment regrowth kinetics of each MTS. That is, if a spheroid regrows slowly, its 'growth delay' will appear longer than that of another spheroid which has a faster regrowth but whose cell number has been reduced to the same extent by the treatment. On the other hand, calculations of log kill based on the Gompertz growth model are acceptable because the assumptions listed in Material and methods are valid in our experimental set-up. We believe that this is indeed the case because the ITs are endowed with biological and pharmacological properties satisfying those assumptions: in particular ITs (1) kill cells following first-order kinetics and (2) exert their cytotoxic effects very rapidly (few hours) compared with the whole post-treatment growth kinetics of the MTSs (days).

Our results show that the individual response of tumour micromasses to anti TfnR-IT and to ricin toxin is extremely heterogeneous. Whereas it is not surprising to observe considerable differences in sensitivity to IT between monolayers and MTSs (Chignola *et al.*, 1994*b*), it was rather unexpected to find such large differences among tumour micromasses obtained with the same cell line and treated at the same IT concentrations. We ruled out the possibility that the heterogeneity in response to treatment of individual MTSs could be due to uncertainties in the Gompertzian analysis. In fact:

1. An analogous degree of heterogeneity was found when experimental data on the growth delay induced by the treatments were considered. Values of the growth delay can be extrapolated from Figures 8 and 9. For both MCF7 and 9L MTSs the growth delay was highly heterogeneous, varying between 0 days and 8.0 days.
2. The surviving fraction of cells calculated with the Gompertzian method, when plotted against the growth delay (Figures 8 and 9), provided results which are in agreement with theoretical predictions and paralleling findings obtained with independent analytical methods reported by other investigators (Twentyman, 1980).
3. Also, the 'all or nothing' effect shown by IT or toxin at given concentrations (see, for example, Figure 2c) can be qualitatively appreciated. Therefore, the heterogeneous response of MTSs to IT treatment appears to be the consequence of biological differences between individual MTSs.

The heterogeneity of response to treatment of individual MTSs might be due to several factors probably acting in concert (e.g. extracellular matrix composition, permeability and retention kinetics of IT molecules within individual MTSs, etc.), which will be investigated in future studies. Nonetheless, at least some factors could be demonstrated not to be of relevance in our model on the basis of the following results:

1. The heterogeneous response of MTSs to ricin treatment rules out inhomogeneous or insufficient expression of target antigens as a cause of low sensitivity. In fact, ricin binds galactose and *N*-acetylgalactosamine residues present in very high numbers at the cell surface (Nicholson and Blaustein, 1972; Olsnes and Pihl, 1982). We cannot however, exclude the possibility that in other instances heterogeneous expression of target antigen may result in widely differing effects on three-dimensional structures.
2. Heterogeneity can be observed with both MCF7 and 9L MTSs treated with anti TfnR-IT; MCF7 (sensitive) and 9L (resistant) cells were used because they show two distinct patterns of sensitivity to anti TfnR-IT (Chignola *et al.*, 1994*b*). Thus, intrinsic sensitivity of the target cells to the pharmacological agents used in our assays does not explain the wide range of cytoreductive effects observed by us in MTSs of both cell lines.

The results in Figures 8 and 9 indicate that, although treated non-sterilised MTSs and control MTSs reach the same final limiting volumes, treated MTSs approach this upper limit following different individual growth patterns. In fact, for the same surviving fraction of cells, a wide range of doubling times could be estimated. Growth kinetics heterogeneities have been found to affect chemotherapy (Norton, 1985). Moreover, we have recently found that the IT cytoreductive effects may be heavily influenced by the proliferative potential of the target cells (Chignola *et al.*, 1994*a*). The results shown in the present report suggest that this might indeed be the case for MTSs treated with anti-TfnR IT and with ricin toxin. The scatter of post-treatment growth kinetics parameters (epitomised by the heterogeneity of $T_D$ values in surviving MTSs) indicates that (1) different MTSs are composed of different numbers of IT-resistant cells, which regrow after treatment following variable kinetics, or (2) the IT treatment facilitates the escape of cell populations endowed with different growing potential. Further efforts in elucidating the relationship between cell subpopulations within individual tumour masses and sensitivity to IT treatment are therefore warranted the better to evaluate the IT anti-tumour potential.

To our knowledge, the cytoreductive effects brought about in MTS cultures by other therapeutic agents do not result in such heterogeneous effects, although occasionally variable log kill effects are reported for radiation and chemotherapeutic agents (examples in Twentyman, 1980; Sutherland, 1988; Walker *et al.*, 1988; Rofstad and Sutherland, 1989; Kikuchi *et al.*, 1992). The higher heterogeneity observed with IT may be explained in terms of the more physiological conditions used in our assays to estimate the log kill or with the macromolecular nature of our reagents.

Our study was designed to investigate the effects of IT treatments on three-dimensional tumour structures sharing many characteristics with tumour micrometastases *in vivo* (Sutherland, 1988). We show that at suboptimal concentrations the cytoreductive effects of the IT are unpredictably heterogeneous. We also show that treated non-sterilised MTSs regrow until their volume reaches the same upper volume as control MTSs, demonstrating that non-sterilising treatments do not result in a stable reduction of the tumour cell burden. This may have important consequences for the therapeutic use of IT *in vivo*, effective IT concentrations at the site of the tumour, or within it, being reduced by a combination of systemic and local factors. It must be considered, however, that even at suboptimal IT concentrations we could obtain substantial cytoreduction in many instances. In our experiments only one treatment schedule was investigated (one round, 24 h treatment). We cannot rule out the possibility that other more complex schedules (e.g. several rounds, longer treatments) could have resulted in less heterogeneous effects or in greater cytoreduction. The MTS model may be helpful in the future for optimising the conditions required to achieve greater IT potency against solid tumour structures.

**Abbreviations:** IT, immunotoxin; MTS, multicellular tumour spheroid; RTA, ricin toxin A chain; CRM107, diphtheria toxin mutant; Tfn, transferrin; TfnR, transferrin receptor; HSA-Mo, thioether-based conjugate between monensin and human serum albumin; FBS, fetal bovine serum; PBS, phosphate-buffered saline; BSA, bovine serum albumin.

**Acknowledgements**

This work was supported by grants from CNR, (PF Ingegneria Genetica, PF Applicazioni Cliniche della Ricerca Oncologica), Associazione Italiana per la Ricerca sul Cancro (AIRC), Murst 40% Aspetti Clinico Sperimentali della Risposta Immune, MS ISS Progetto AIDS, Associazione per la Promozione delle Ricerche Biomediche (ARBI), Murst 60%, Murst 40% Neuroimmunologia. R Chignola is a recipient of an ISS fellowship. M Pasti is a recipient of an AIRC fellowship. The expert technical help of E Chiesa and M Tommasi is gratefully acknowledged. We thank Dr RJ Youle for comments and suggestions.

**References**

BAIRD Y. (1974). *Non-linear Parameter Estimation*. Academic Press: New York.

BRUNTON GF AND WHELDON TE. (1980). The Gompertz equation and the construction of tumor growth curves. *Cell Tissue Kinet.*, **13**, 455-460.

CANDIANI C, FRANCESCHI A, CHIGNOLA R, PASTI M, ANSELMI C, BENONI G, TRIDENTE G AND COLOMBATTI M. (1992). Blocking effect of human serum but not of cerebrospinal fluid on ricin A chain immunotoxin potentiaion by monensin or carrier protein-monensin conjugates. *Cancer Res.*, **52**, 623-630.

CHEN FM, HANSEN EB, TAYLOR CR AND EPSTEIN AL. (1991). Diffusion and binding of monoclonal antibody TNT-1 in multicellular tumor spheroids. *J. Natl Cancer Inst.*, **83**, 200-204.

CHIGNOLA R, ANSELMI C, FRANCESCHI A, PASTI M, CANDIANI C, TRIDENTE G AND COLOMBATTI M. (1994*a*). Sensitivity of human leukemia cells in exponential or stationary growth phase to anti-CD5 immunotoxins. Role of processing events. *J. Immunol.*, **152**, 2333-2343.

CHIGNOLA R, FORONI R, CANDIANI C, FRANCESCHI A, PASTI M, STEVANONI G, ANSELMI C, TRIDENTE G AND COLOMBATTI M. (1994*b*). Cytoreductive effects of anti-transferrin receptor immunotoxins in a multicellular tumor spheroid model. *Int. J. Cancer*, **57**, 268-274.

DEMICHELI R. (1980). Growth of testicular neoplasm lung metastases: tumor specific relation between two gompertzian parameters. *Eur. J. Cancer*, **16**, 1603-1608.

DEMICHELI R, FORONI R, GIULIANI F AND SAVI G. (1988). Influence of tumor growth kinetics on response to doxorubicin treatment of C3H mammary carcinoma. *Tumori*, **74**, 269-274.

DEMICHELI R, FORONI R, INGROSSO A, PRATESI G, SORANZO C AND TORTORETO M. (1989). An exponential-gompertzian description of LoVo cell tumor growth from in vivo and in vitro data. *Cancer Res.*, **49**, 6543-6546.

DEMICHELI R, PRATESI G AND FORONI R. (1991). The exponential-Gompertzian tumor growth model: data from six tumor cell lines in vitro and in vivo. Estimate of the transition point from exponential to gompertzian growth and potential clinical implications. *Tumori*, **77**, 189-195.

JOHNSON VG, WROBEL C, WILSON D, ZOVICKIAN J, GREENFIELD L, OLDFIELD EH AND YOULE RJ. (1989). Improved tumor-specific immunotoxins in the treatment of CNS and leptomeningeal neoplasia. *J. Neurosurg.*, **70**, 240-248.

KIKUCHI T, OHNUMA T, HOLLAND JF AND SPITLER LE. (1992). Penetration of anti-melanoma immunotoxins into multicellular tumor spheroids and cell kill effects. *Cancer Immunol. Immunother.*, **35**, 302-306.

KWOK CS, COLE SE AND LIAO SK. (1988). Uptake kinetics of monoclonal antibodies by human malignant melanoma multicell spheroids. *Cancer Res.*, **48**, 1856-1863.

LANDAW EM AND DISTEFANO III JJ. (1984). Multiexponential, multicompartmental and non-compartmental modeling. II. Data analysis and statistical considerations. *Am. J. Physiol.*, **246**, R665-R677.

LLOYD HH. (1975). Estimation of tumor cell kill from Gompertz growth curves. *Cancer Chemother. Rep.*, **59**, 267-277.

NICHOLSON GL AND BLAUSTEIN J. (1972). The interaction of *Ricinus communis* agglutinin with normal and tumor cell surfaces. *Biochim. Biophys. Acta*, **266**, 543-547.

NORTON L. (1985). Implications of kinetic heterogeneity in clinical oncology. *Semin. Oncol.*, **12**, 231-249.

OLSNES S AND PIHL A. (1982). Chimeric toxins. *Pharmacol. Ther.*, **15**, 355-381.

ROFSTAD EK AND SUTHERLAND RM. (1989). Growth and radiation sensitivity of the MLS human ovarian carcinoma cell line as multicellular spheroids and xenografted tumors. *Br. J. Cancer*, **59**, 28-35.

SKIPPER HE, SCHABEL JR FM AND WILCOX WS. (1964). Experimental evaluation of potential anticancer agents. XIII. On the criteria and kinetics associated with 'curability' of experimental leukemia. *Cancer Chemother. Rep.*, **35**, 1-111.

SUTHERLAND RM. (1988). Cell and environment interactions in tumor microregions: the multicell spheroid model. *Science*, **240**, 177-184.

TWENTYMAN PR. (1980). Response to chemotherapy of EMT6 spheroids as measured by growth delay and cell survival. *Br. J. Cancer*, **42**, 297-304.

VITETTA ES, THORPE PE AND UHR JW. (1993). Immunotoxins: magic bullets or misguided missiles? *Immunol. Today*, **14**, 252-259.

WALKER KA, MURRAY T, HILDITCH TE, WHELDON TE, GREGOR A AND HANN IM. (1988). A tumor spheroid model for antibody-targeted therapy of micrometastases. *Br. J. Cancer*, **58**, 13-16.

WEINSTEIN JN AND VAN OSDOL W. (1992). Early intervention in cancer using monoclonal antibodies and other biological ligands: micropharmacology and the 'binding site barrier'. *Cancer Res.*, **52**, 2747s-2751s.

YUHAS JM, LI AP, MARTINEZ AO AND LANDMAN AJ. (1977). A simplified method for the production and growth of multicellular tumor spheroids. *Cancer Res.*, **37**, 3639-3643.